# Ubiquitous filter feeders shape open ocean microbial community structure and function

**DOI:** 10.1093/pnasnexus/pgae091

**Published:** 2024-03-19

**Authors:** Anne W Thompson, Györgyi Nyerges, Kylee M Lamberson, Kelly R Sutherland

**Affiliations:** Department of Biology, Portland State University, Portland, OR 97201, USA; Department of Biology, Pacific University, Forest Grove, OR 97116, USA; Department of Chemistry, Portland State University, Portland, OR 97201, USA; Oregon Institute of Marine Biology, University of Oregon, Eugene, OR 97403, USA

**Keywords:** *Crocosphaera*, *Prochlorococcus*, SAR11, grazing, salps

## Abstract

The mechanism of mortality plays a large role in how microorganisms in the open ocean contribute to global energy and nutrient cycling. Salps are ubiquitous pelagic tunicates that are a well-known mortality source for large phototrophic microorganisms in coastal and high-latitude systems, but their impact on the immense populations of smaller prokaryotes in the tropical and subtropical open ocean gyres is not well quantified. We used robustly quantitative techniques to measure salp clearance and enrichment of specific microbial functional groups in the North Pacific Subtropical Gyre, one of the largest ecosystems on Earth. We discovered that salps are a previously unknown predator of the globally abundant nitrogen fixer *Crocosphaera*; thus, salps restrain new nitrogen delivery to the marine ecosystem. We show that the ocean's two numerically dominant cells, *Prochlorococcus* and SAR11, are not consumed by salps, which offers a new explanation for the dominance of small cells in open ocean systems. We also identified a double bonus for *Prochlorococcus*, wherein it not only escapes salp predation but the salps also remove one of its major mixotrophic predators, the prymnesiophyte *Chrysochromulina*. When we modeled the interaction between salp mesh and particles, we found that cell size alone could not account for these prey selection patterns. Instead, the results suggest that alternative mechanisms, such as surface property, shape, nutritional quality, or even prey behavior, determine which microbial cells are consumed by salps. Together, these results identify salps as a major factor in shaping the structure, function, and ecology of open ocean microbial communities.

**Significance Statement**

The vast open oceans are dominated by small bacteria that are interspersed with larger, but less abundant, functionally diverse phytoplankton. We show that selective feeding of ubiquitous pelagic tunicates (salps), contributes to this microbial community structure in several ways. Salps do not retain *Prochlorococcus* and SAR11 during feeding, consume the protistan predators of these small bacteria, maintain low nutrient levels by removing the nitrogen-fixer *Crocosphaera*, and ease competition for nutrients by capturing other primary producers, such as diatoms and *Synechococcus.* We also found that cell size could not completely explain *Prochlorococcus* and SAR11 escape from salp grazing, suggesting novel mechanisms for evading predation by the world's most numerous marine bacteria and a globally important role for salps in shaping microbial community structure in one of Earth's largest ecosystems.

## Introduction

Mortality mechanisms play a large part in determining the specific contributions of marine microorganisms to ecosystems. For example, carbon from a marine microorganism will be recycled upon viral lysis (1), delivered to higher trophic levels if consumed by nanoflagellates (2), or exported to the deep sea if incorporated into sinking particles (3, 4). Therefore, knowledge of specific mortality mechanisms, beyond overall loss rates, is a key to understanding the different ways marine microorganisms contribute to global processes.

Recent work has advanced quantitative understanding of the rates, dynamics, and selectivity of viral lysis and protistan predation (5–7), the most well-understood microbial mortality sources in the oceans. However, when this understanding is brought together, predictions of mortality do not always match observations (8–13). Thus, there are likely other key microbial predators that remain poorly understood. Quantifying predation rates and selectivity for powerful but poorly understood predators is of utmost importance to closing this gap and producing accurate models of open ocean microbial communities and their dynamic interactions with higher trophic levels.

Salps are one of the most understudied microbial predators in the surface ocean. These pelagic tunicates are ubiquitous filter feeders that capture a range of particles by pumping seawater through mucous mesh nets. Salp feeding can export microbial carbon to the deep sea as sinking fecal pellets (3, 14–17) or “short-circuit” food webs by transferring microbial carbon to top trophic levels in just a few predation interactions (18–20). Salp feeding may also be selective among microbial prey, where microbes with similar properties (e.g. size) are consumed nonuniformly (21–25). This selective feeding may influence microbial community composition, size structure, and direct and indirect interactions between microorganisms. However, nearly all existing studies of salp predation on marine microorganisms are focused on high latitude or coastal systems where microbial biomass is dominated by relatively large eukaryotic microbial taxa (3, 24). These systems contrast with the small-cell-dominated open-ocean gyres that cover much of Earth (26) and harbor previously overlooked pelagic tunicate biomass (27). Earlier studies relied on microscopy (28), coulter counters (29), or were based on cultured prey (30) or microspheres (31) and therefore could not account for small cells, especially those <1 µm. More recent studies that have been conducted in small-cell dominated systems lack robustly quantitative approaches and precise microbial taxonomy that would yield conclusive insight into feeding selectivity between coexisting microorganisms (21–23). Thus, a major gap exists in understanding the role of pelagic tunicates in microbial mortality within large open ocean gyres.

In this study, we examined the predation of salps on several functionally distinct and coexisting microbial taxa in the vast oligotrophic North Pacific Subtropical Gyre (NPSG). We used blue-water diving techniques to quantify clearance rates (CRs) and feeding-organ enrichment of the different microbial prey precisely (i.e. with highly sensitive flow cytometry and qPCR). We also modeled the interaction between spherical particles and salp mesh to identify whether particle size or some other physical mechanism governs salp selective feeding on these microorganisms. These results identify salps as an important control on microbial community size structure, function, and productivity of the open ocean gyres.

## Results

### Salp and microbial species sampled in the oligotrophic open ocean

**Fig. 1.**
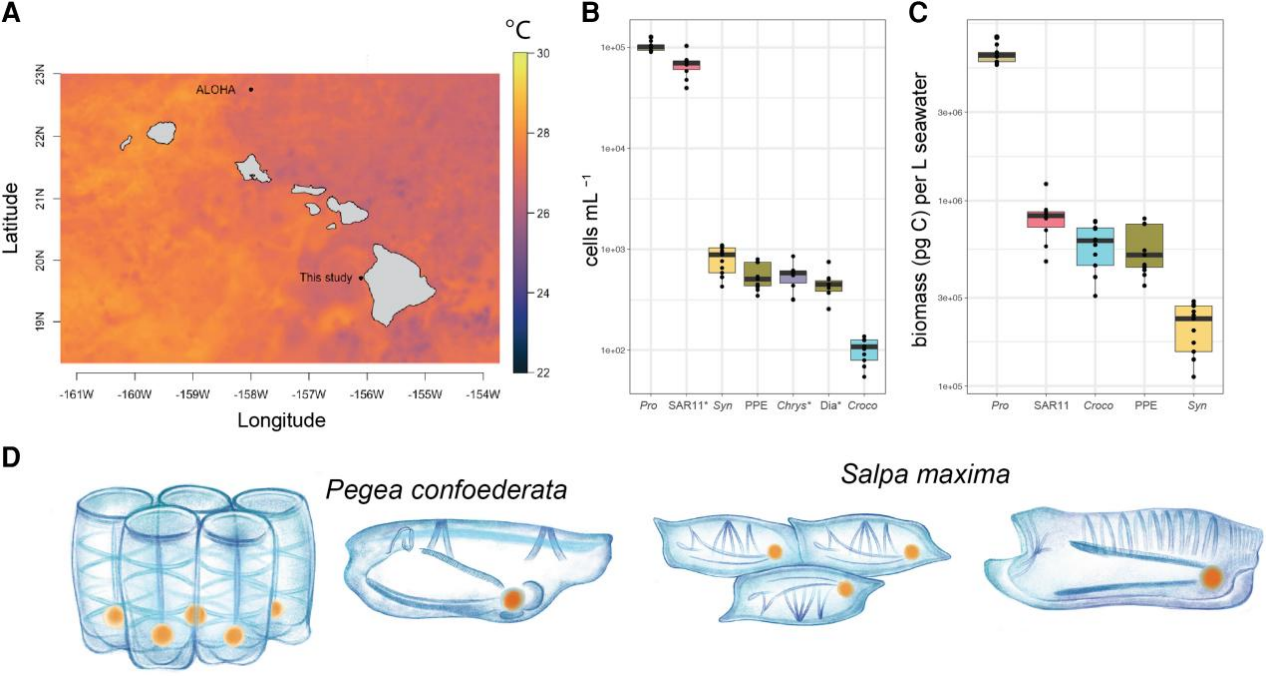
A) Sea surface temperature at the sampling site in September 2022 and proximity to Station ALOHA, an oceanographic station representative of the North Pacific Subtropical Gyre. B) Concentration of microbial cells (cells per mL) determined by flow cytometry or qPCR (denoted *). Counts from qPCR were corrected if organisms had multiple gene copies per cell, as described in the Materials and methods section. C) Biomass contributions (pg C) of microorganisms with known per-cell carbon quotas (Table S2). D) Illustrations of studied salps in solitary and colonial forms (by Franz Anthony). *Croco*, *Crocosphaera* sp.; *Chrys*, *Chrysochromulina*; Dia, diatoms; *Pro*, *Prochlorococcus*; *Syn*, *Synechococcus*.

Our sampling station was offshore of the island of Hawai’i, chosen for its narrow shelf region (Fig. 1A) and access to deep open-ocean waters representative of the NPSG (32). Regional satellite-derived sea surface temperatures during field sampling (September 2022) matched station ALOHA, a representative site for the NPSG (33). The satellite-derived sea temperatures at the study site (∼26–28 °C) corresponded to in situ measurements (26–27 °C) during SCUBA dives within the top 25 m of the surface ocean.

The community structure and abundances of marine microorganisms we encountered were consistent with previous observations at Station ALOHA (13, 34–36) and the broader region (37–39). *Prochlorococcus* and SAR11 numerically dominated the microbial community, with counts exceeding other picocyanobacteria (*Synechococcus*), nitrogen-fixing cyanobacteria (*Crocosphaera*), and pigmented picoeukaryotes (PPEs), including diatoms and the mixotrophic prymnesiophyte *Chrysochromulina* by 2–3 orders of magnitude (Fig. 1B). Despite its small size (40), *Prochlorococcus* dominated the microbial biomass of the system, followed by the PPEs and *Crocosphaera*. *Synechococcus* and SAR11 provided the lowest biomass contributions (Fig. 1C).

We encountered a wide range of pelagic tunicates during blue-water SCUBA dives at the field site. For experimentation and collection, we targeted common and abundant open ocean salp species, including *Pegea confoederata* and *Salpa maxima* (Fig. 1D). Free-swimming salps were identified at the moment of live capture. Both solitary and aggregate (i.e. colonial) life stages were included in the study.

### CRs on phytoplankton

To quantify the impact of salp feeding on open ocean microbial communities, we began by measuring the CRs of different coexisting salp taxa on phytoplankton that we could enumerate by flow cytometry. We performed feeding incubations with 13 live salps (*P. confoederata*, *n* = 9 and *S. maxima*, *n* = 4) alongside 13 nonanimal controls. While all salps pumped seawater throughout the incubations, we were unable to visually confirm the presence of feeding meshes through the incubation jars.

**Fig. 2.**
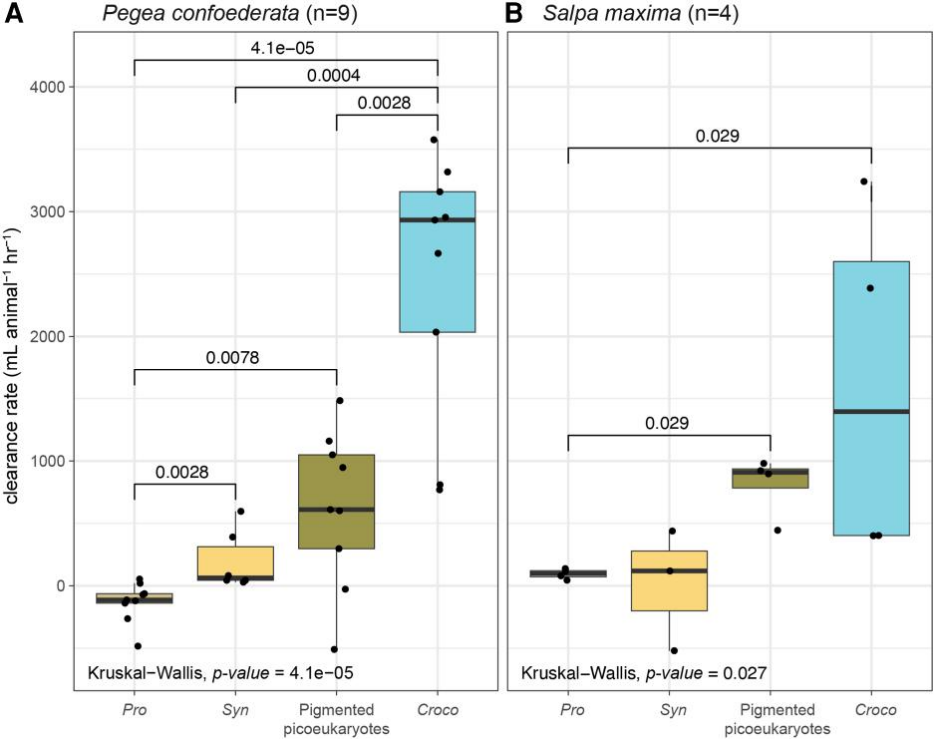
Clearance rates of each salp type, A) *Pegea confoederata* and B) *Salpa maxima,* on each phytoplankton prey type detected by flow cytometry. The number of salps tested is indicated by *n*. For boxplots, the middle line is the median, the top line is the 75th percentile, and the bottom line is the 25th percentile. Whiskers extend to the largest and the smallest values within the interquartile range. Dots are the individual data points (i.e. each salp). The overall test of differences by sample type was performed with Kruskal–Wallis. *Croco*, *Crocosphaera* sp.; *Pro*, *Prochlorococcus*; *Syn*, *Synechococcus*.

We observed significant differences in CRs between the microbial prey types (Fig. 2). *Crocosphaera* was cleared at rates higher than the other phytoplankton (*P* < 0.01), with medians of about 3,000 mL (*P. confoederata*) and 2,000 mL (*S. maxima*) of seawater per animal per hour. In some cases, *P. confoederata* and *S. maxima* removed all *Crocosphaera* from the incubation bottles within an hour (Fig. S1), suggesting that CRs in an unrestricted prey field (i.e. the ocean) would be even higher. PPE CRs were lower than *Crocosphaera* (*P* < 0.01 *P. confoederata*, not significant *S. maxima* due to low *n*) but exceeded the picocyanobacteria. Among the picocyanobacteria, *Synechococcus* clearance rates exceeded *Prochlorococcus* (*P* = 0.0028 *P. confoederata*, n.s. *S. maxima* due to low *n*). *Prochlorococcus* CRs were at or below zero, indicating no significant difference from the no-animal controls. CRs for the individual phytoplankton prey were negatively correlated to cell concentrations in the prey field (Fig. S2) and positively correlated to predicted cell diameters (Fig. S5).

### Biomass contributions of cleared phytoplankton

**Fig. 3.**
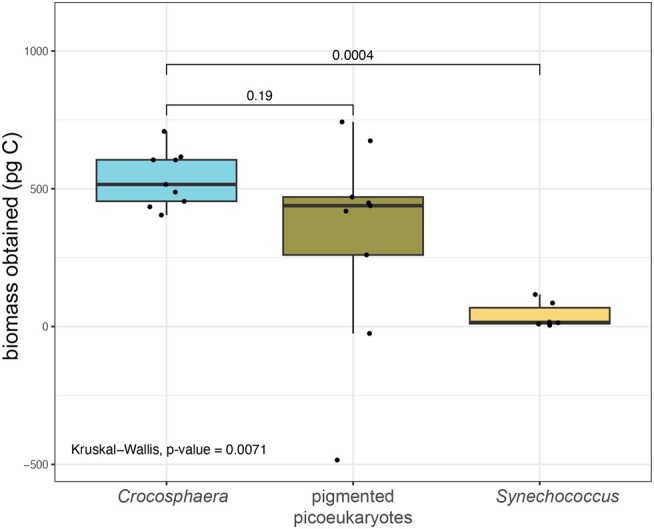
Biomass contributions of prey populations consumed by *P. confoederata* calculated from the number of cells removed during the live incubations and their per-cell carbon quotas (Table S2). For boxplots, the middle line is the median, the top line is the 75th percentile, and the bottom line is the 25th percentile. Whiskers extend to the largest and the smallest values within the interquartile range. Dots are the individual data points for each incubated salp. The overall test of differences by sample type was performed with Kruskal–Wallis.

To discern the contribution of each microbial lineage to the total phytoplankton biomass consumed by the salps, we calculated the biomass of the cells removed from the *P. confoederata* incubations using the CRs to derive the number of cells removed and per-cell carbon quotas from previous works for these organisms in this system (Table S2). We found that the PPEs and *Crocosphaera* contributed the same amount of carbon to the salps (*P* > 0.05), which exceeded the very low average contributions of *Synechococcus* (*P* < 0.01; Fig. 3). As *Prochlorococcus* CRs were not different from zero, biomass contributions were not calculated.

### Gut enrichment of diverse major microbial functional groups

To broaden the microbial taxa we could study, differentiate between functional groups, and quantitatively test for feeding selectivity, we used qPCR to quantify the gene copy number (i.e. cell number) of several dominant microbial taxa in dissected salp guts. Given its strong feeding signal, and numerous replicates collected, we focused on *P. confoederata* for the qPCR analysis. The prey quantities in guts were compared with seawater, all normalized by volume, and corrected for multiple gene copies per cell, to calculate the enrichment of each microbial taxa in the salp guts relative to seawater.

**Fig. 4.**
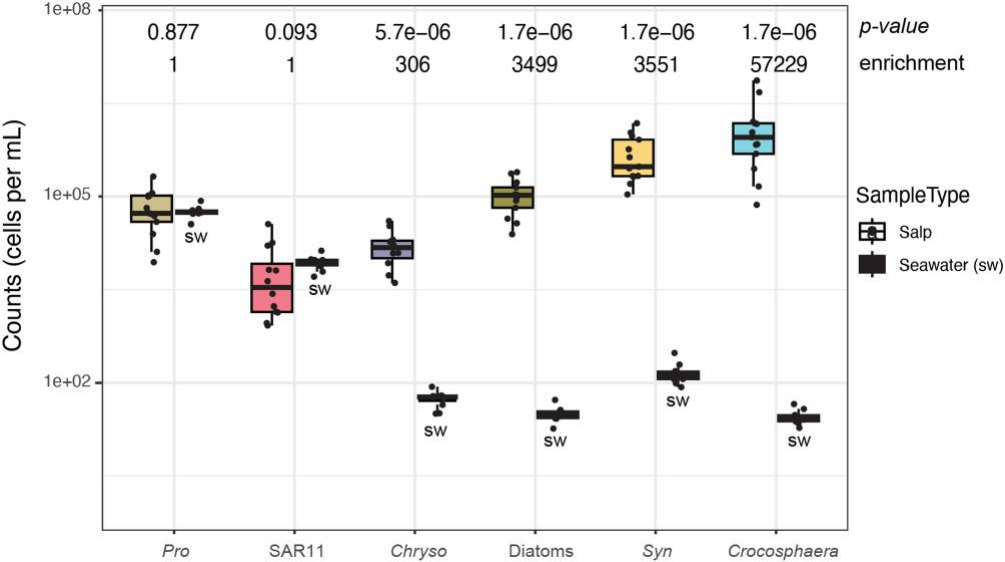
Concentration (cells per mL) of prey in each *P. confoederata* gut compared with surrounding seawater as determined by qPCR using RA to reduce inhibition. Counts from qPCR were corrected if organisms had multiple gene copies per cell, as described in the Materials and methods section, to get cells per volume. The significance of the differences between salp and seawater for each qPCR assay (*x*-categories) is indicated by its *P-*value and the fold enrichment in the salp gut relative to seawater (enrichment). For boxplots, the middle line is the median, the top line is the 75th percentile, and the bottom line is the 25th percentile. Whiskers extend to the largest and the smallest values within the interquartile range. Dots are the individual salps tested for each qPCR assay. The overall test of differences by sample type was performed with Kruskal–Wallis. Seawater abundances are less here than in Fig. 1, because we used RA in all qPCRs to reduce inhibition by the salp guts and wanted to allow a fair comparison between gut and seawater amplification (see Materials and methods). *Chryso*, *Chrysochromulina*; *Pro*, *Prochlorococcus*; *Syn*, *Synechococcus*.

We found dramatic differences in how enriched the different microbial prey were in the salp guts compared with surrounding seawater (Fig. 4). *Prochlorococcus* and SAR11 were not different between the seawater and *P. confoederata* guts (*P* > 0.05), indicating no enrichment in the gut compared with the same volume of seawater. All other microbial taxa were significantly enriched in the salp guts relative to seawater (*P* << 0.01). *Crocosphaera* cells were most enriched at over 57,000-fold more abundant in the guts than the same volume of seawater. *Synechococcus* and the diatoms were enriched about 3,500-fold in *P. confoederata* guts compared with the same volume of seawater (*P* < 0.01). *Chrysochromulina* was enriched about 300-fold in the salp guts compared with seawater (*P* < 0.01).

### Pegea particle encounter predictions

**Fig. 5.**
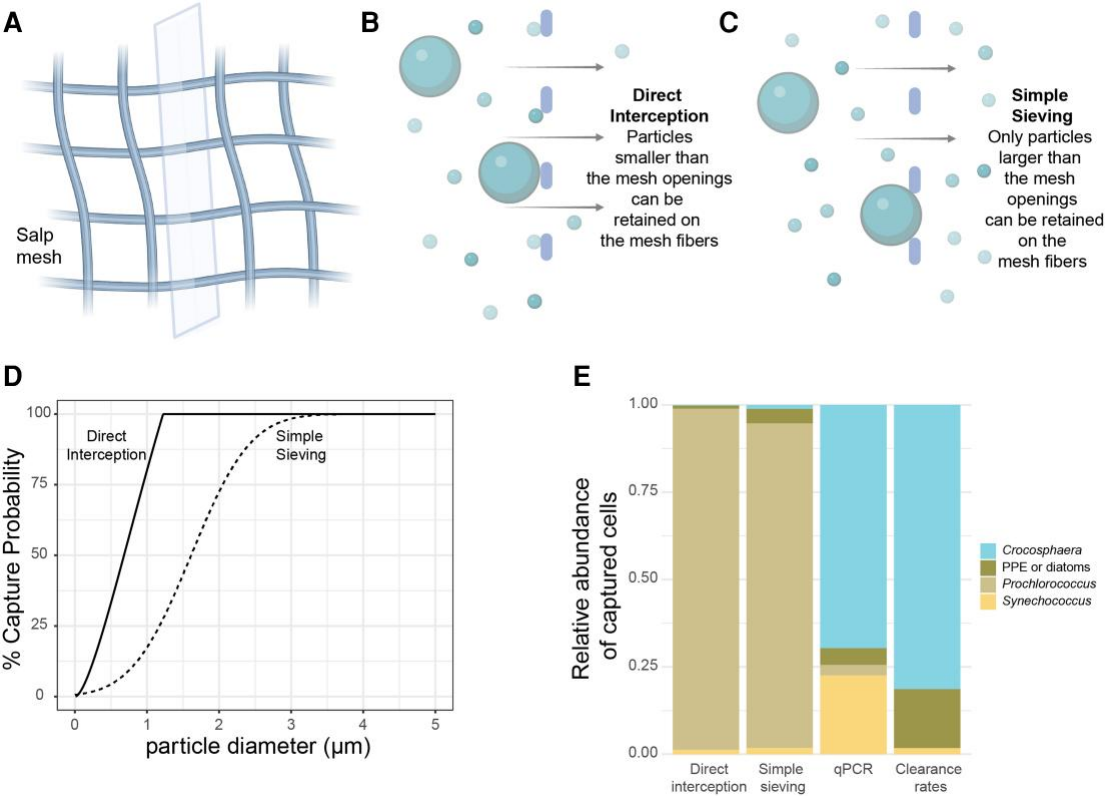
Investigation of two different models of particle encounter between salp mesh and microorganisms. A) Illustration of salp mesh after microscopy images published in Sutherland et al. (18) with mesh fiber cross-section (gray rectangle) to indicate the perspective of B and C. B) Visualization of direct interception looking at cross-sections of the salp mesh fibers (gray), with arrows indicating the path of fluid flow, and spheres indicating particles of different sizes. C) Visualization of simple sieving as in B. D) Particle capture probabilities based on direct interception and simple sieving with a Gaussian mesh for *P. confoederata*. E) Comparison of the relative abundance of retained prey (colors) from theoretical particle encounter models (direct interception vs. simple sieving), results from qPCR analysis, and CRs from flow cytometry analysis. For the qPCR data, diatom abundance is shown as one component of the PPEs. Predicted particle encounter efficiencies and predicted counts of captured cells are presented in Fig. S3.

To identify whether size is the mechanism by which salps retain some microbial prey and pass over others, we compared theoretical model predictions of *P. confoederata* particle encounter under two different scenarios: either direct interception or simple sieving of spherical particles (Fig. 5A–C). The direct interception scenario is a low-Reynolds (low-*Re*) number hydrodynamics model based on classic aerosol filtration theory (41). At sufficiently small length scales and low velocities, low-*Re* filtration theory predicts that flow separation around filter elements will be minimal and particles smaller than the mesh openings can be directly intercepted on mesh fibers. The simple sieving scenario assumes that any particles larger than the mesh openings will be captured, where the mesh openings are described by a Gaussian distribution around the mean mesh opening size. The direct interception model predicts that particles >1.2 µm will be captured with 100% efficiency and the simple sieving model predicts that particles >∼3 µm will be captured with 100% efficiency (Fig. 5D).

Both models predicted patterns directly counter to the enrichment and CR empirical results. Predicted capture was highest for *Prochlorococcus*, followed by *Synechococcus* and PPEs, while predicted capture of *Crocosphaera* was negligible (Fig. 5E). The model of simple sieving showed overall slightly lower capture efficiency and encounter rates than those predicted by the direct interception model (Fig. S3A and B). While these models accurately predicted the capture of inert spherical particles of different sizes in a previous study with the same salp species (*P. confoederata*) (18), we show that the models do not accurately predict live cell capture. Collectively, these results suggest that simple mechanical principles—particle size, mesh dimensions, and flow rates through the mesh—are not sufficient to explain cell capture by salps. Instead, other properties of the mucous mesh and/or microbes, including shape, surface properties, nutritional quality, or behavior may be governing capture.

## Discussion

This work examined salps as a mortality source for the large microbial populations that dominate the Earth's open ocean gyres. Using classic blue-water SCUBA techniques to ensure undisturbed feeding (42, 43), we measured CRs on diverse microbial prey. Using taxa-specific molecular assays, we compared specific prey in their enrichment level in salp guts relative to surrounding seawater. To understand the mechanism by which certain microbial groups escape grazing, we compared model-derived prey profiles from different particle encounter scenarios to our results. We found that salps play a unique role in the biogeochemical function and ecosystem structure of the open oceans through selective feeding on different microbial functional groups.

### Salps are a previously unknown predator of *Crocosphaera*

*Crocosphaera* stood out from the other microbial taxa due to its high CRs by both salp taxa, high enrichment in salp guts, and high contributions to prey biomass. This is the first evidence that mucous mesh grazers are the predators of *Crocosphaera*. As a ubiquitous cell adapted to widespread ocean regimes (44, 45), especially the nutrient poor open ocean, our finding has important implications for how *Crocosphaera* contributes to marine food webs and biogeochemical cycles on global scales.

The identification of pelagic tunicates as *Crocosphaera* predators adds new complexity to the understanding of *Crocosphaera* loss processes and their consequences, which are only just beginning to be addressed. Viruses are one possible mortality source for *Crocosphaera* (46). While the details of the viral lysis of *Crocosphaera* have yet to be revealed, we hypothesize that viral lysis releases unique dissolved organic matter to the surrounding seawater as it does for other microbial cells (1). However, at the moment, the bulk of *Crocosphaera* mortality is attributed to a variety of protists, which range in size from 6 to 30 µm, and include mixotrophs (38). Culture work suggests diel dynamics in protistan grazing on *Crocosphaera* (47), potentially influencing the rhythm of nutrient supply to the microbial loop through sloppy feeding. In contrast to viral lysis and protistan sloppy feeding, *Crocosphaera* captured by salps could be propelled to the highest trophic levels when salps are predated by seabirds and sea turtles (48) or exported to the deep ocean through sinking fecal pellets, as salps and other pelagic tunicates do for phytoplankton in higher latitude systems (3, 4, 17). Indeed, sinking particulates from the summer export pulse at Station ALOHA contain *Crocosphaera* (49), suggesting a role for salps in exporting phytoplankton biomass in the oligotrophic open ocean as well as at high latitudes. A direct comparison of these mortality sources will be important to expand our understanding of the ecosystem consequences of *Crocosphaera* loss.

Removal of *Crocosphaera* by salps also has important repercussions for biogeochemical pathways in the surface ocean. As a diazotroph, *Crocosphaera* delivers new nitrogen to low nutrient environments, supporting diverse microbial life across huge areas of the ocean and playing a globally important role in the nitrogen cycle (39, 44). This versatile cell can also incorporate fixed nitrogen, competing against nondiazotrophic phytoplankton and heterotrophic microorganisms for nitrogen species, such as ammonium (50). These nitrogen metabolisms, especially the nitrogen-fixation machinery, come with a very high iron demand, which *Crocosphaera* is uniquely adapted to manage despite very low iron availability in open ocean gyres (51, 52). With these adaptations, *Crocosphaera* contributes to net primary production in the subtropical gyres on the same scale as the more abundant PPEs at around 11% of the net community production (34, 38, 39). Thus, in the removal of *Crocosphaera*, salps restrict the amount of new nitrogen that enters the surface ocean, limit productivity, and may direct these macro- and micronutrients to the deep sea through their fast-sinking fecal pellets or to higher trophic levels rather than to the microbial loop. Salp predation on *Crocosphaera* also brings more complexity to the interplay of iron availability, atmospheric carbon dioxide concentrations, and nitrogen fixation in future climate scenarios (53, 54).

### The ocean's dominant cells evade grazing by ubiquitous predators

This study is the first to show in a robustly quantitative way that *Prochlorococcus* and SAR11 evade grazing by open ocean salps. While a previous study reported that nonsalp mucous filter feeders capture *Prochlorococcus* but not SAR11 (21), that conclusion relied on amplicon sequence data, which is compositional and thus ambiguous in inferring changes in absolute cell abundances between samples (55–58). Two other studies also reported low retention of *Prochlorococcus* by salps, but *Prochlorococcus* identification was not definitive due to a low sensitivity flow cytometry method (22) or uncertain identification with microscopy (24). In summary, while previous works have alluded to this finding before, ours is the first study that combines precise microbial identification with a statistically rigorous approach to show that open ocean salps do not feed on *Prochlorococcus* and SAR11.

*Prochlorococcus* and SAR11 evasion of salp grazing in these experiments adds to the building evidence that protists and viruses are the key predators of these dominant bacterial lineages (5, 7, 36, 59–61). This finding will improve parameterization of all of these predators (zooplankton, protists, and viruses) in ecosystem models (9). Further, the insight into SAR11 and *Prochlorococcus* predator avoidance has implications for the structure and evolution of marine microbial communities.

*Prochlorococcus* and SAR11 escape from salp grazing also relate to a long-standing question of why the tropical and subtropical open ocean gyres are dominated by small cells. This question is especially relevant to climate science as small marine cells may flourish in future climate scenarios (62–64) and to fundamental questions in biology as the Earth's most numerous cells are small. Bottom-up explanations, such as the theory of streamlining (65, 66) and the temperature-size rule (67, 68), are powerful in explaining why small cells thrive in the warm nutrient-poor open ocean. Our work complements these two bottom-up explanations of the small-cell dominance phenomenon. First, our work shows that salps remove larger cells from the ocean system through selective feeding, possibly through selection based on more than size, leaving populations of smaller cells unchanged. Second, while untested, it is also possible that SAR11 and *Prochlorococcus* small size is an adaptive trait used to escape predation by large mucous mesh feeders. Though patchy in their distribution, mucous mesh feeders are constantly present in the open ocean and have formidable filtration rates (19). In the study area near the Hawaiian Islands, salp biomass ranges from 100 to 1,000 mg C m^−3^, representing some of the highest global salp biomasses (69). In the oligotrophic Atlantic Ocean (Sargasso Sea), mean salp abundances are 3.32 ± 25.5 ind m^−3^, reaching a maximum of 281 ind m^−3^ (70). Collectively, these data point to salps as an important source of mortality in the oligotrophic ocean. For SAR11, their slippery membranes may work together with their small cell size as an adaptative strategy to avoid pelagic tunicates and ascidians (21).

This story becomes more complicated because it is expected that small size makes cells more susceptible to protist predation (71). Thus, in escaping mucous mesh grazers, *Prochlorococcus* and SAR11 may be more susceptible to small predators, such as nanoflagellates and ciliates. Given that *Prochlorococcus* and SAR11 likely coevolved in the early ocean (72), the avoidance of salps by both taxa may tie into their shared evolutionary trajectory. While many open questions remain on this topic, our work makes it clear that these cells must tread a fine line to balance their size, nutrient acquisition efficiency, and vulnerability to a range of predator sizes and strategies.

### Salps shape direct and indirect microbial interactions

The qPCR approach enabled direct study of known microbial functional groups; thus, we can infer how salps influence organismal interactions relevant to the open ocean ecosystem.

We discovered several direct interactions that would be altered by salp feeding. In quantifying salp removal of *Chrysochromulina*, a known *Prochlorococcus* predator (36, 59), we show that salp feeding may lessen this protist's predation on *Prochlorococcus* by reducing the abundance of the predator population. Given that *Prochlorococcus* escapes the salp predation too, salp predation on *Chrysochromulina* is a double bonus for *Prochlorococcus*. Salp feeding may also alter direct competition between open ocean phytoplankton. By capping the population sizes of larger phytoplankton, salps may reduce direct competition between phytoplankton for scarce nutrients in the oligotrophic oceans.

Our work also shows that salps influence indirect interactions between microorganisms, which are difficult interactions to detect, but are key layers in microbial community dynamics and function. We reveal that salps are shared predators of *Synechococcus*, diatoms, and mixotrophic phytoplankton, meaning that salp growth from feeding on one of these phytoplankton groups will increase mortality of the other groups, which can seem like competition between the phytoplankton (a.k.a. apparent competition) (73). This insight is important for parameterizing predator–prey relationships in ecosystem models. In particular, recent work shows that incorporation of shared predation into models of marine bacteria biogeography improves model accuracy and alignment with ship-based observations (12).

### Factors other than cell size govern escape

Our results from natural prey populations are in direct opposition to capture rates predicted by two theoretical models. Models of direct interception and simple sieving filtration suggest that even though capture efficiencies are low (57 and 8%, respectively, Figs. 5 and S3) for small particles like *Prochlorococcus*, they are predicted to be captured at high rates due to their numerical abundance. Conversely, large particles like *Crocosphaera* are predicted to be captured at close to 100% efficiency but represent a very low proportion of the salp diet due to their low abundances in the seawater. Intriguingly, previous incubation experiments with *P. confoederata* grazing different-sized artificial polystyrene microspheres matched the results from the low-*Re* number particle capture model (18); therefore, differences between model and experiment observed here are likely not due to shortcomings of the model but instead should be attributed to aspects of natural particles other than size that mediate particle capture. This phenomenon has also been observed in benthic ascidians (74), where polystyrene beads were captured much less efficiently than planktonic cells, building evidence that properties such as cell surface may be important in prey capture by filter feeders.

Existing understanding of these open ocean microorganisms provides some hypotheses for what cell properties could govern the predation interaction. Particle shape has been shown to influence capture (75) but this effect is insufficient to account for different capture rates in the present study. For example, *Prochlorococcus* and *Synechococcus* are similar in size and both roughly spherical but they experienced divergent capture rates in this study (Figs. 2 and 4), consistent with previous studies of gelatinous grazers (23). Surface properties of particles, including hydrophobicity, have also been shown to mediate particle capture (74, 76). In particular, members of the SAR11 clade have a lower hydrophobicity index than other dominant microbes and have been shown to evade capture by some benthic and pelagic tunicates (21). Though untested, *Prochlorococcus* may similarly evade capture due to cell surface attributes. Further, the behavior of salps or the prey may also dictate capture. Salps can swim backward and eject the feeding mesh to reject particles in bulk (77) but this behavior does not account for more nuanced particle selection. Behavioral responses to predators are almost completely unknown and unexplored for the small prey cells in this study, but bacteria from other systems are known to have varied tools of defense (78–80).

In conclusion, this work shows a major role for salps in controlling the abundances and collective function of microbial communities in the vast nutrient-poor open ocean. By removing larger microorganisms, salps alter the encounters between small bacteria, their competitors, and their protistan predators. Comparison of our results to different models of particle retention on mucous mesh filters suggests that unknown cell properties, such as surface characteristics, behavior, and shape, may play a role in determining which cells are captured by salps and which cells escape to populate and thrive in one of the Earth's most expansive ecosystems.

## Materials and methods

### Oceanographic setting and sampling

Collections and experiments were conducted in the North Pacific Subtropical Gyre, at a field site 3 nautical miles offshore of Kona, Hawai’i (19.710746 N, 22.75 W; Fig. 1A). This site was chosen for its position within the North Pacific Subtropical Gyre, and close proximity to the long-term Station ALOHA, which is strongly representative of this oceanic region (32). Satellite-measured sea surface temperatures were analyzed retrospectively with the Group for High Resolution Sea Surface Temperature Level 4 using version 4 Multiscale Ultrahigh Resolution (MUR) L4 analysis at the beginning of the field sampling period (2022 September 11). The data were obtained from the Physical Oceanography Distributed Active Archive Center (PODAAC; https://podaac.jpl.nasa.gov/MEaSUREs-MUR).

All samplings were conducted from a recreational dive boat using blue-water SCUBA techniques (43). Dives were conducted just prior to sunset or immediately after sunset to have the opportunity to sample different salps at different times in case of shifting community composition due to vertical migration (81). Sample collection occurred in the top 25 m.

### Salp and prey field collections

To capture salps for incubations or gut dissections, divers approached salps slowly and enclosed them in 1 L jars. We used this method to limit disturbance to the salp feeding process, such as ejection or cessation of feeding net production. For salps intended for incubations, an initial sample (“time zero”) of an undisturbed parcel of seawater (i.e. the prey field) near the animal was collected in a 3-mL syringe immediately after animal capture. Negative controls for the incubations were treated the same as the salp, without the capturing of an animal. Within 15 min of collection, divers surfaced and passed the collection jars and time-zero samples to a person on the dive boat deck, who continued the incubations, sampling at several intervals across the time-course, or performed the gut dissections.

### Feeding incubations

Feeding incubations were initiated at the point of collection (time zero) and finished on board the dive boat. Incubations were sheltered from direct sunlight or deck lights (for after dark incubations) and lasted from 45 to 90 min. The incubation jar lids were custom-fitted with self-healing rubber injection ports (13 mm diameter, Ks-Tek), allowing sample collection using a needle and syringe without disturbing the animals. Samples for flow cytometry were collected at regular intervals (about every 30 min) in 1 mL volumes fixed with a final concentration of 0.125% electron microscopy grade glutaraldehyde (Tousimis, Rockville, MD, USA). Fixed samples were gently inverted 10 times, then incubated at ambient temperature in the dark. Samples were stored on blue ice (∼0 °C) until the dive boat returned to shore when samples were frozen on dry ice then archived at −80 °C.

### Gut dissections and volume estimates

Additional *P. confoederata* were collected to quantify prey within the gut. Within 30 min of collection, the salps were poured gently from the collection jar onto a mesh sieve and gently rinsed three times with 0.2 µm filtered seawater from the site. Dissection tools were sterilized in ethanol and treated with Thermo Scientific DNA AWAY (Thermo Fisher Scientific, Waltham, MA, USA) before each use. Dissecting scissors were used to cut the gut out of the animal and place it in a sterile bead-beater tube preloaded with 100 µL DNA/RNA Shield (Zymo Research, Irvine, CA, USA). Gut volumes were estimated at 100 µL, based on their diameter and estimation as a sphere.

### Flow cytometry

In the live salp incubations, the predicted size and concentrations of *Prochlorococcus*, *Synechococcus*, PPEs, and *Crocosphaera* were quantified via flow cytometry using a BD Influx high-speed cell sorted (BD Biosciences, San Jose, CA, USA) equipped with a small particle detector. Cells were excited by a 488-nm laser with a trigger on forward light scatter (FSC). Yellow-green, 1-µm-diameter polystyrene beads (Polysciences, Inc., Warrington, PA, USA) were used for laser alignment, size calibration, and as a reference for gating. The volume of each sample analyzed was calculated from the time analyzed and flow rate of the instrument. Gating of the phytoplankton populations was done in *FlowJo* version 10.4.1 (BD Biosciences), following many previous works in this system (13, 82) and recent descriptions of marine phytoplankton populations commonly detected by flow cytometry (83). The Mie theory was used to estimate the average equivalent spherical diameter (ESD) for each population of cells detected via flow cytometry, following previous work that validated this approach on phytoplankton in the NPSG (84). First, we calculated the optimized Mie model for the BD Influx instrument used in this study using a range of spherical particles. We measured mean FSC for each population (Fig. S4A) then, following Ribalet et al. (84), we estimated the average ESD of each phytoplankton population based on the instrument-specific Mie relationship and the FSC means (Fig. S4B).

### Biomass calculations

We estimated the biomass contributed by each microbial group detected via flow cytometry and selected groups detected by qPCR (i.e. SAR11). Microbial population biomass (pg C L^−1^) was calculated by multiplying previously published per-cell carbon quotas by the number of cells per volume (measured in this study via flow cytometry or qPCR). Table S2 presents the values and references for all carbon cell quota values. While there are per cell carbon quotas published for relatives of *Chrysochromulina* and *Thalassiosira* (84–86), we did not compute biomass estimates for these two taxa, due to the lack of published values on strains of these lineages acclimated to open ocean conditions, which could be significantly different from strains in culture.

### Quantitative PCR

The qPCR was used to quantify six different microbial prey types in the DNA from dissected guts of *P. confederata* and surrounding seawater (50 mL volumes filtered on 0.2 µm pore-sized filters). Targets included *Prochlorococcus* eMIT9312, SAR11, marine *Synechococcus* (clade 2), *Crocosphaera*, diatom *Thalassiosira* sp., and haptophyte *Chrysochromulina*. This approach follows work developed for the quantification of eukaryotic phytoplankton in doliolids (87, 88). We corrected gene copies per mL detected by qPCR to cells per mL based on copy number per cell, which is 1 for all bacterial assays, 2 for *Chrysochromulina* (36), and 1 for the diatom (with the caveat that diatom 18S rRNA gene copy number per cell can vary substantially across taxa, as described (89)). We found substantial qPCR inhibition by the salp DNA, which informed many of the subsequent steps. Seawater DNA did not inhibit qPCRs.

DNA was extracted from seawater filters and from salp guts using the DNeasy Plant Tissue Mini kit or the Blood and Tissue kit (Qiagen, Hilden, Germany) with some modifications to the manufacturer's protocol. We added three cycles of freezing the samples in liquid nitrogen for 30 s and thawing them at 65 °C before the TissueLyser step, followed by a proteinase K treatment before the RNase treatment step. DNA was eluted in 100 µL of “AE” buffer. The two kits resulted in the same DNA yields. We found no difference in how DNA from the two different extraction methods inhibited PCR by using a universal internal positive control (IPC) to quantify inhibition, as previously published (90). To minimize PCR inhibition, we also used the OneStep PCR Inhibitor Removal kit (Zymo Research) for the salp gut DNA samples.

We performed the qPCRs using either the Power SYBR Green PCR Master Mix (Applied Biosystems, Waltham, MA, USA) or the TaqMan Universal Master Mix (Thermo Fisher Scientific), depending on the published assay protocols (Table S1). We had 10 seawater DNA samples and 13 *P. confoederata* gut DNA samples. Standard curves for each assay were created between 10^0^ and 10^6^ gene copies using gBlocks synthetic oligonucleotides (Integrated DNA Technologies, Coralville, IA, USA) in Tris-EDTA or “AE” buffer, following published recommendations (91). Oligonucleotide sequences used for the standards are provided in Table S2. All reactions were run in duplicates or triplicates, and at least two no-template controls were used in each qPCR run. At the end of each run, a melt curve analysis confirmed that only one amplicon was produced. The qPCRs were run on either a MiniOpticon System (Bio-Rad Laboratories, Hercules, CA, USA) or on a ViiA7 Real-time PCR system (Thermo Fisher Scientific) in 20 µL volume.

To further minimize inhibition, we diluted (1:5) the salp DNA to reduce the presence of inhibitors and added 1 µL of 20 mg/mL recombinant albumin (RA; New England Biolabs, Beverley, MA, USA) to each qPCR reaction (1 mg/mL final concentration). When RA was used in the reactions, the same amount was added to the standard curve reactions. We confirmed that these treatments eliminated inhibition using an IPC (90). However, we noticed that while eliminating inhibition, the RA reduced the maximum fluorescence of double-stranded DNA in the qPCRs. Previous studies indicated that while albumin can counteract polymerization inhibitors, it does not eliminate the quenching of fluorescence (92). Furthermore, in the IPC reactions with RA, we consistently observed lower fluorescence and a small but consistent increase in cycle number (Cq). In order to ensure that we did not introduce any difference between the salp and seawater DNA gene copy numbers with the method, we ran a subset of seawater DNA samples with RA. We calculated a conversion factor based on the difference in Cq between reactions with and without RA and applied this conversion to the rest of the seawater DNA samples. For the abundances of microbial taxa in seawater (Fig. 1), we report qPCR values run without RA for the best qPCR runs. However, because the salp guts had to be analyzed with the RA, when we compared gut microbial prey with seawater, we used seawater values with RA (Fig. 4).

### Clearance rates calculations

We calculated the CRs, or volume swept clear, of salps on the different microbial prey that were detected by flow cytometry in the live incubations using the particle depletion method (93). This method has often been used for salps (94), including the study target *P. confoederata* (95). CR was calculated, as detailed previously (96), using the formula:


$$\mathrm{CR}=\left(\frac{V}{nt}\right)\ln\;\left(\frac{C_{c}}{C_{e}}\right),$$

where *C*_c_ and *C*_e_ are the concentration of prey cells at the end of the incubation in the no-animal control (*C*_c_) and with-animal experiment (*C*_e_) after time (*t*), the volume of the incubation (*V*), and number of animals (*n*; always one).

### Particle encounter model

To tease apart potential physical mechanisms that govern differential CRs, and enrichment, of natural microbial prey populations, we calculated capture efficiencies (*E*, dimensionless), using two theoretical models. First, a low-*Re* number hydrodynamics model based on direct interception of particles on a rectangular mesh was used with field measured parameters (particle sizes) or parameters taken from the literature (*P. confoederata* mesh dimensions) following previous studies (18, 97). Particles were assumed to be spherical and particles that contacted the mesh were assumed to stick to the mesh. Second, a model of simple sieving was used based on a previously determined Gaussian distribution of mesh widths for *P. confoederata* (18). The Gaussian distribution accounts for the observed variability in mesh opening size such that particles that are smaller than the mean mesh opening can still be retained. Capture efficiency, *E*, was then used to determine an encounter rate, *P* (particles s^−1^) from each model following:


P=EQC,

where *Q* (mL/s) is the average volume flow rate through a 40-mm long salp—set to *Q* = 1.69 mL/s based on empirical measurements from three previous studies (described in Sutherland et al. (18))—and *C* (particles mL^−1^) is the particle concentration measured directly from the field samples. The encounter rate was then used to compare model-predicted capture to empirical measurements.

## Supplementary Material

pgae091_Supplementary_Data

## Data Availability

All data are available in the main text and Supplementary material.

## References

[1] Ma X , Coleman ML, Waldbauer JR. 2018. Distinct molecular signatures in dissolved organic matter produced by viral lysis of marine cyanobacteria. Environ Microbiol. 20:3001–3011. 30047191 10.1111/1462-2920.14338

[2] Jürgens K , Wickham SA, Rothhaupt KO, Santer B. 1996. Feeding rates of macro- and microzooplankton on heterotrophic nanoflagellates. Limnol Oceanogr. 41:1833–1839.

[3] Décima M , et al 2023. Salp blooms drive strong increases in passive carbon export in the Southern Ocean. Nat Commun. 14:425. 36732522 10.1038/s41467-022-35204-6 PMC9894854

[4] Jaspers C , et al 2023. Gelatinous larvacean zooplankton can enhance trophic transfer and carbon sequestration. Trends Ecol Evol. 38:980–993. 37277269 10.1016/j.tree.2023.05.005

[5] Carlson MCG , et al 2022. Viruses affect picocyanobacterial abundance and biogeography in the North Pacific Ocean. Nat Microbiol. 7:570–580. 35365792 10.1038/s41564-022-01088-x PMC8975747

[6] Connell PE , Ribalet F, Armbrust EV, White A, Caron DA. 2020. Diel oscillations in the feeding activity of heterotrophic and mixotrophic nanoplankton in the North Pacific Subtropical Gyre. Aquat Microb Ecol. 85:167–181.

[7] Landry MR , Stukel MR, Selph KE, Goericke R. 2023. Coexisting picoplankton experience different relative grazing pressures across an ocean productivity gradient. Proc Natl Acad Sci U S A. 120:e2220771120. 10.1073/pnas.2220771120 PMC10622918 37871180

[8] Talmy D , et al 2019. An empirical model of carbon flow through marine viruses and microzooplankton grazers. Environ Microbiol. 21:2171–2181. 30969467 10.1111/1462-2920.14626

[9] Talmy D , Carr E, Rajakaruna H, Vage S, Willem-Omta A. 2023. Killing the predator: impacts of top predator mortality on global-ocean ecosystem structure. Biogeosciences Discuss. [preprint]. doi:10.5194/bg-2023-120 [in review, 2023].

[10] Beckett SJ , et al Diel population dynamics and mortality of Prochlorococcus in the North Pacific Subtropical Gyre. biorxiv 448546. 10.1101/2021.06.15.448546, preprint: not peer reviewed. PMC10920773 38453897

[11] Rajakaruna H , Omta AW, Carr E, Talmy D. 2023. Linear scaling between microbial predator and prey densities in the global ocean. Environ Microbiol. 25:306–314. 36335554 10.1111/1462-2920.16274 PMC10100078

[12] Follett CL , et al 2022. Trophic interactions with heterotrophic bacteria limit the range of Prochlorococcus. Proc Natl Acad Sci U S A. 119:e2110993118. 10.1073/pnas.2110993118 PMC8764666 34983874

[13] Ribalet F , et al 2015. Light-driven synchrony of *Proclorococcus* growth and mortality in the subtropical Pacific gyre. Proc Natl Acad Sci U S A. 112:8008–8012. 26080407 10.1073/pnas.1424279112 PMC4491802

[14] Silver MW , Bruland KW. 1981. Differential feeding and fecal pellet composition of salps and pteropods, and the possible origin of the deep-water flora and olive-green “cells”. Mar Biol. 62:263–273.

[15] Dubischar CD , Bathmann UV. 1997. Grazing impact of copepods and salps on phytoplankton in the Atlantic sector of the Southern Ocean. Deep Sea Res Part II Top Stud Oceanogr. 44:415–433.

[16] Henschke N , Everett JD, Richardson AJ, Suthers IM. 2016. Rethinking the role of salps in the ocean. Trends Ecol Evol. 31:720–733. 27444105 10.1016/j.tree.2016.06.007

[17] Steinberg DK , et al 2023. The outsized role of salps in carbon export in the subarctic Northeast Pacific Ocean. Glob Biogeochem Cycles. 37:e2022GB007523. 10.1029/2022GB007523 PMC10078299 37034114

[18] Sutherland KR , Madin LP, Stocker R. 2010. Filtration of submicrometer particles by pelagic tunicates. Proc Natl Acad Sci U S A. 107:15129–15134. 20696887 10.1073/pnas.1003599107 PMC2930554

[19] Sutherland KR , Thompson AW. 2022. Pelagic tunicate grazing on marine microbes revealed by integrative approaches. Limnol Oceanogr. 67:102–121.

[20] Cavallo C , et al 2018. Molecular analysis of predator scats reveals role of salps in temperate inshore food webs. Front Mar Sci. 5:381.

[21] Dadon-Pilosof A , et al 2017. Surface properties of SAR11 bacteria facilitate grazing avoidance. Nat Microbiol. 2:1608–1615. 28970475 10.1038/s41564-017-0030-5

[22] Dadon-Pilosof A , Lombard F, Genin A, Sutherland KR, Yahel G. 2019. Prey taxonomy rather than size determines salp diets. Limnol Oceanogr. 64:1996–2010.

[23] Thompson AW , Sweeney CP, Sutherland KR. 2023. Selective and differential feeding on marine prokaryotes by mucus mesh feeders. Environ Microbiol. 25:880–893. 36594240 10.1111/1462-2920.16334

[24] Stukel MR , Décima M, Selph KE, Gutiérrez-Rodríguez A. 2021. Size-specific grazing and competitive interactions between large salps and protistan grazers. Limnol Oceanogr. 66:2521–2534.

[25] Fender CK et al Prey Size Spectra and Predator:Prey ratios of 7 Species of New Zealand Salps. biorxiv 480784. 10.1101/2022.02.16.480784, preprint: not peer reviewed.

[26] Karl DM , Bidigare RR, Letelier RM. 2001. Long-term changes in plankton community structure and productivity in the North Pacific Subtropical Gyre: the domain shift hypothesis. Deep Sea Res Part II Top Stud Oceanogr. 48:1449–1470.

[27] Luo JY , Stock CA, Henschke N, Dunne JP, O’Brien TD. 2022. Global ecological and biogeochemical impacts of pelagic tunicates. Prog Oceanogr. 205:102822.

[28] Vargas CA , Madin LP. 2004. Zooplankton feeding ecology: clearance and ingestion rates of the salps *Thalia democratica*, *Cyclosalpa affinis* and *Salpa cylindrica* on naturally occurring particles in the Mid-Atlantic Bight. J Plankton Res. 26:827–833.

[29] Deibel D . 1985. Blooms of the pelagic tunicate, *Dolioletta gegenbauri*: are they associated with Gulf Stream frontal eddies? J Mar Res. 43:211–236.

[30] Harbison GR , McAlister VL. 1979. The filter-feeding rates and particle retention efficiencies of three species of *Cyclosalpa* (Tunicata, Thaliacea). Limnol Oceanogr. 24:875–892.

[31] Kremer P , Madin LP. 1992. Particle retention efficiency of salps. J Plankton Res. 14:1009–1015.

[32] Dore JE , Church MJ, Karl DM, Sadler DW, Letelier RM. 2014. Paired windward and leeward biogeochemical time series reveal consistent surface ocean CO2 trends across the Hawaiian Ridge. Geophys Res Lett. 41:6459–6467.

[33] Karl DM , Lukas R. 1996. The Hawaii Ocean Time-series (HOT) program: background, rationale and field implementation. Deep-Sea Res Part II Top Stud Oceanogr. 43:129–156.

[34] Rii YM , Karl DM, Church MJ. 2016. Temporal and vertical variability in picophytoplankton primary productivity in the North Pacific Subtropical Gyre. Mar Ecol Prog Ser. 562:1–18.

[35] Karl DM , Church MJ. 2014. Microbial oceanography and the Hawaii ocean time-series programme. Nat Rev Micro. 12:699–713. 10.1038/nrmicro3333 25157695

[36] Li Q , Edwards KF, Schvarcz CR, Steward GF. 2022. Broad phylogenetic and functional diversity among mixotrophic consumers of Prochlorococcus. ISME J. 16:1557–1569. 35145244 10.1038/s41396-022-01204-z PMC9122939

[37] Gradoville MR , et al 2020. Latitudinal constraints on the abundance and activity of the cyanobacterium UCYN-A and other marine diazotrophs in the North Pacific. Limnol Oceanogr. 65:1847–1857.

[38] Dugenne M , Henderikx Freitas F, Wilson ST, Karl DM, White AE. 2020. Life and death of Crocosphaera sp. in the Pacific Ocean: fine scale predator–prey dynamics. Limnol Oceanogr. 65:2603–2617.

[39] Wilson ST , et al 2017. Coordinated regulation of growth, activity and transcription in natural populations of the unicellular nitrogen-fixing cyanobacterium Crocosphaera. Nat Microbiol. 2:17118. 28758990 10.1038/nmicrobiol.2017.118

[40] Cermak N , et al 2017. Direct single-cell biomass estimates for marine bacteria via Archimedes’ principle. ISME J. 11:825–828. 27922599 10.1038/ismej.2016.161 PMC5322313

[41] Rubenstein DI , Koehl MA. 1977. The mechanisms of filter feeding: some theoretical considerations. Am Nat. 111:981–994.

[42] Hamner WM , Madin LP, Alldredge AL, Gilmer RW, Hamner PP. 1975. Underwater observations of gelatinous zooplankton: sampling problems, feeding biology, and behavior. Limnol Oceanogr. 20:907–917.

[43] Haddock SH , Heine JN. 2005. Scientific blue-water diving. La Jolla (CA): California Sea Grant College Program, University of California.

[44] Moisander PH , et al 2010. Unicellular cyanobacterial distributions broaden the oceanic N2 fixation domain. Science. 327:1512–1514. 20185682 10.1126/science.1185468

[45] Webb EA , Ehrenreich IM, Brown SL, Valois FW, Waterbury JB. 2009. Phenotypic and genotypic characterization of multiple strains of the diazotrophic cyanobacterium, *Crocosphaera watsonii*, isolated from the open ocean. Environ Microbiol. 11:338–348. 19196268 10.1111/j.1462-2920.2008.01771.x

[46] Luo E , Eppley JM, Romano AE, Mende DR, DeLong EF. 2020. Double-stranded DNA virioplankton dynamics and reproductive strategies in the oligotrophic open ocean water column. ISME J. 14:1304–1315. 32060418 10.1038/s41396-020-0604-8 PMC7174320

[47] Deng L , Cheung S, Liu H. 2020. Protistal grazers increase grazing on unicellular Cyanobacteria diazotroph at night. Front Mar Sci. 7:135.

[48] Cardona L , Quevedo IÁ, de Borrell A, Aguilar A. 2012. Massive consumption of gelatinous plankton by Mediterranean apex predators. PLoS One. 7:e31329. 22470416 10.1371/journal.pone.0031329 PMC3310041

[49] Karl DM , Church MJ, Dore JE, Letelier RM, Mahaffey C. 2012. Predictable and efficient carbon sequestration in the North Pacific Ocean supported by symbiotic nitrogen fixation. Proc Natl Acad Sci U S A. 109:1842–1849. 22308450 10.1073/pnas.1120312109 PMC3277559

[50] Masuda T , et al 2022. Crocosphaera as a major consumer of fixed nitrogen. Microbiol Spectr. 10:e02177–e02121. 35770981 10.1128/spectrum.02177-21 PMC9431459

[51] Saito MA , et al 2011. Iron conservation by reduction of metalloenzyme inventories in the marine diazotroph *Crocosphaera watsonii*. Proc Natl Acad Sci U S A. 108:2184–2189. 21248230 10.1073/pnas.1006943108 PMC3038740

[52] Webb EA , Moffett JW, Waterbury JB. 2001. Iron stress in open-ocean Cyanobacteria (*Synechococcus*, *Trichodesmium*, and *Crocosphaera* spp.): identification of the IdiA protein. Appl Environ Microbiol. 67:5444–5452. 11722891 10.1128/AEM.67.12.5444-5452.2001 PMC93328

[53] Fu F-X , et al 2008. Interactions between changing pCO2, N2 fixation, and Fe limitation in the marine unicellular cyanobacterium Crocosphaera. Limnol Oceanogr. 53:2472–2484.

[54] Yang N , et al 2022. Molecular mechanisms underlying iron and phosphorus co-limitation responses in the nitrogen-fixing Cyanobacterium *Crocosphaera*. ISME J. 16:2702–2711. 36008474 10.1038/s41396-022-01307-7 PMC9666452

[55] McMurdie PJ , Holmes S. 2014. Waste not, want not: why rarefying microbiome data is inadmissible. PLoS Comput Biol. 10:e1003531. 24699258 10.1371/journal.pcbi.1003531 PMC3974642

[56] Tsilimigras MCB , Fodor AA. 2016. Compositional data analysis of the microbiome: fundamentals, tools, and challenges. Ann Epidemiol. 26:330–335. 27255738 10.1016/j.annepidem.2016.03.002

[57] Gloor GB , Macklaim JM, Pawlowsky-Glahn V, Egozcue JJ. 2017. Microbiome datasets are compositional: and this is not optional. Front Microbiol. 8:2224. 29187837 10.3389/fmicb.2017.02224 PMC5695134

[58] Weiss S , et al 2017. Normalization and microbial differential abundance strategies depend upon data characteristics. Microbiome. 5:27. 28253908 10.1186/s40168-017-0237-y PMC5335496

[59] Frias-Lopez J , Thompson A, Waldbauer J, Chisholm SW. 2009. Use of stable isotope-labelled cells to identify active grazers of picocyanobacteria in ocean surface waters. Environ Microbiol. 11:512–525. 19196281 10.1111/j.1462-2920.2008.01793.x PMC2702499

[60] Orsi WD , et al 2018. Identifying protist consumers of photosynthetic picoeukaryotes in the surface ocean using stable isotope probing. Environ Microbiol. 20:815–827. 29215213 10.1111/1462-2920.14018

[61] Våge S , Storesund JE, Thingstad TF. 2013. SAR11 viruses and defensive host strains. Nature. 499:E3–E4. 23887434 10.1038/nature12387

[62] Bian V , Cai M, Follett CL. 2023. Understanding opposing predictions of Prochlorococcus in a changing climate. Nat Commun. 14:1445. 36922531 10.1038/s41467-023-36928-9 PMC10017810

[63] Morán XAG , et al 2015. More, smaller bacteria in response to ocean's warming? Proc R Soc B Biol Sci. 282:20150371. 10.1098/rspb.2015.0371 PMC4590472 26063843

[64] Flombaum P , Martiny AC. 2021. Diverse but uncertain responses of picophytoplankton lineages to future climate change. Limnol Oceanogr. 66:4171–4178.

[65] Giovannoni SJ , Thrash JC, Temperton B. 2014. Implications of streamlining theory for microbial ecology. ISME J. 8:1553–1565. 24739623 10.1038/ismej.2014.60 PMC4817614

[66] Zehr JP , Weitz JS, Joint I. 2017. How microbes survive in the open ocean. Science. 357:646–647. 28818930 10.1126/science.aan5764

[67] Atkinson D . 1994. Temperature and organism size-a biological law for ectotherms? Adv Ecol Res. 25:1–58.

[68] Forster J , Hirst AG, Esteban GF. 2013. Achieving temperature-size changes in a unicellular organism. ISME J. 7:28–36. 22832346 10.1038/ismej.2012.76 PMC3526166

[69] Lucas CH , et al 2014. Gelatinous zooplankton biomass in the global oceans: geographic variation and environmental drivers. Glob Ecol Biogeogr. 23:701–714.

[70] Stone JP , Steinberg DK. 2016. Salp contributions to vertical carbon flux in the Sargasso Sea. Deep Sea Res Part Oceanogr Res Pap. 113:90–100.

[71] Boenigk J , Stadler P, Wiedlroither A, Hahn MW. 2004. Strain-specific differences in the grazing sensitivities of closely related ultramicrobacteria affiliated with the Polynucleobacter cluster. Appl Environ Microbiol. 70:5787–5793. 15466515 10.1128/AEM.70.10.5787-5793.2004 PMC522116

[72] Braakman R , Follows MJ, Chisholm SW. 2017. Metabolic evolution and the self-organization of ecosystems. Proc Natl Acad Sci U S A. 114:E3091–E3100. 28348231 10.1073/pnas.1619573114 PMC5393222

[73] Holt RD , Bonsall MB. 2017. Apparent competition. Annu Rev Ecol Evol Syst. 48:447–471.

[74] Jacobi Y , et al 2021. Evasive plankton: size-independent particle capture by ascidians. Limnol Oceanogr. 66:1009–1020.

[75] Conley KR , Sutherland KR. 2017. Particle shape impacts export and fate in the ocean through interactions with the globally abundant appendicularian *Oikopleura dioica*. PLoS One. 12:e0183105. 28854260 10.1371/journal.pone.0183105 PMC5576645

[76] Monger BC , Landry MR, Brown SL. 1999. Feeding selection of heterotrophic marine nanoflagellates based on the surface hydrophobicity of their picoplankton prey. Limnol Oceanogr. 44:1917–1927.

[77] Harbison GR , McAlister VL, Gilmer RW. 1986. The response of the salp, *Pegea confoederata*, to high levels of particulate material: starvation in the midst of plenty. Limnol Oceanogr. 31:371–382.

[78] Mazzola M , de Bruijn I, Cohen MF, Raaijmakers JM. 2009. Protozoan-induced regulation of cyclic lipopeptide biosynthesis is an effective predation defense mechanism for *Pseudomonas fluorescens*. Appl Environ Microbiol. 75:6804–6811. 19717630 10.1128/AEM.01272-09 PMC2772446

[79] Song C , et al 2015. Molecular and chemical dialogues in bacteria-protozoa interactions. Sci Rep. 5:12837. 26246193 10.1038/srep12837 PMC4542665

[80] Wildschutte H , Wolfe DM, Tamewitz A, Lawrence JG. 2004. Protozoan predation, diversifying selection, and the evolution of antigenic diversity in Salmonella. Proc Natl Acad Sci U S A. 101:10644–10649. 15247413 10.1073/pnas.0404028101 PMC489988

[81] Madin LP , Kremer P, Hacker S. 1996. Distribution and vertical migration of salps (Tunicata, Thaliacea) near Bermuda. J Plankton Res. 18:747–755.

[82] Thompson AW , *et al*. 2018. Dynamics of Prochlorococcus diversity and photoacclimation during short-term shifts in water column stratification at Station ALOHA. Front Mar Sci. 5:488. doi:10.3389/fmars.2018.00488.

[83] Thyssen M , et al 2022. Interoperable vocabulary for marine microbial flow cytometry. Front Mar Sci. 9:975877.

[84] Ribalet F , et al 2019. SeaFlow data v1, high-resolution abundance, size and biomass of small phytoplankton in the North Pacific. Sci Data. 6:277. 31757971 10.1038/s41597-019-0292-2 PMC6874581

[85] Ishiwata Y , Ohi N, Obata M, Taguchi S. 2013. Carbon to volume relationship of *Isochrysis galbana* (Prymnesiophyceae) during cell divisions. Plankton Benthos Res. 8:178–185.

[86] Menden-Deuer S , Lessard EJ. 2000. Carbon to volume relationships for dinoflagellates, diatoms, and other protist plankton. Limnol Oceanogr. 45:569–579.

[87] Frischer ME , et al 2014. Reliability of qPCR for quantitative gut content estimation in the circumglobally abundant pelagic tunicate *Dolioletta gegenbauri* (Tunicata, Thaliacea). Food Webs. 1:18–24.

[88] Frischer ME , et al 2021. Selective feeding and linkages to the microbial food web by the doliolid *Dolioletta gegenbauri*. Limnol Oceanogr. 66: 1993–2010

[89] Gong W , Marchetti A. 2019. Estimation of 18S gene copy number in marine eukaryotic plankton using a next-generation sequencing approach. Front Mar Sci. 6:219.

[90] Kavlick MF . 2018. Development of a universal internal positive control. BioTechniques. 65:275–280. 30394127 10.2144/btn-2018-0034

[91] Conte J , Potoczniak MJ, Tobe SS. 2018. Using synthetic oligonucleotides as standards in probe-based qPCR. BioTechniques. 64:177–179. 29661012 10.2144/btn-2018-2000

[92] Sidstedt M , Rådström P, Hedman J. 2020. PCR inhibition in qPCR, dPCR and MPS—mechanisms and solutions. Anal Bioanal Chem. 412:2009–2023. 32052066 10.1007/s00216-020-02490-2 PMC7072044

[93] Frost BW . 1972. Effects of size and concentration of food particles on the feeding behavior of the marine planktonic copepod calanus Pacificus1. Limnol Oceanogr. 17:805–815.

[94] Madin LP , Kremer P. 1995. Determination of the filter-feeding rates of salps (Tunicata, Thaliacea). ICES J Mar Sci. 52:583–595.

[95] Harbison GR , Gilmer RW. 1976. The feeding rates of the pelagic tunicate *Pegea confoederata* and two other salps1. Limnol Oceanogr. 21:517–528.

[96] Riisgård HU . 2001. On measurement of filtration rates in bivalves—the stony road to reliable data: review and interpretation. Mar Ecol Prog Ser. 211:275–291.

[97] Silvester NR . 1983. Some hydrodynamic aspects of fitter feeding with rectangular-mesh nets. J Theor Biol. 103:265–286.

[98] Brand A , Allen L, Altman M, Hlava M, Scott J. 2015. Beyond authorship: attribution, contribution, collaboration, and credit. Learn Publ. 28:151–155.

