# Supplementary material for: Ubiquitous filter feeders shape open ocean microbial community structure and function
Source: PNAS Nexus. 2024 Mar 19;3(3):pgae091. doi: 10.1093/pnasnexus/pgae091 (PMC10949910; doi:10.1093/pnasnexus/pgae091)
Supplement: pgae091_Supplementary_Data [file pgae091_supplementary_data.pdf]

Supplemental Materials

Ubiquitous filter feeders shape open ocean microbial community structure and function

Anne W. Thompson [1*], Györgyi Nyerges [2], Kylee Brevick [3], Kelly Sutherland [4]

[1] Department of Biology, Portland State University, Portland, OR, 97207.
[2] Pacific University, Department of Biology, Forest Grove, OR, 97116.
[3] Department of Chemistry, Portland State University, Portland, OR, 97207.
[4] University of Oregon, Oregon Institute of Marine Biology, University of Oregon, Eugene, OR 97403.
* Corresponding author

Abstract

The mechanism of mortality plays a large role in how microorganisms of the open ocean contribute to global energy and nutrient cycling. Salps are ubiquitous pelagic tunicates that are a well-known mortality source for large phototrophic microorganisms in coastal and high-latitude systems, but their impact on the immense populations of smaller prokaryotes in the tropical and subtropical open ocean gyres is not well quantified. We used robustly quantitative techniques to measure salp clearance and enrichment of specific microbial functional groups in the North Pacific Subtropical Gyre, one of the largest ecosystems on Earth. We discovered that salps are a previously unknown predator of the globally abundant nitrogen fixer *Crocosphaera*, thus salps restrain new nitrogen delivery to the marine ecosystem. We show that the ocean's two numerically dominant cells, *Prochlorococcus* and SAR11, are not consumed by salps, which offers a new explanation for the dominance of small cells in open ocean systems. We also identified a double bonus for *Prochlorococcus*, wherein it not only escapes salp predation and but the salps remove one of its major mixotrophic predators, the prymnesiophyte *Chrysochromulina*. When we modeled the interaction between salp mesh and particles, we found that cell size alone could not account for these prey selection patterns. Instead, the results suggest that alternative mechanisms such as surface property, shape, nutritional quality, or even prey behavior, determine which microbial cells are consumed by salps. Together these results identify salps as a major factor in shaping the structure, function, and ecology of open ocean microbial communities.

**Supplemental Table 1.** Details of qPCR primers, standards, and cycling conditions.		
Target	Assay Reference(s)	Synthetic oligonucleotide standard sequences (gBlocks)
Thalassiosira sp. (18S rRNA)	(Walters et al. 2019; Frischer et al. 2014)	GAGGTAGTGACAATAAATAACAATGCCGGGCCTTTAC<br>AGGTCTGGCAATTGGAATGAGAACAATTTAAATCCCT<br>TAATCAATTGGAGGGCAAGTCTGGTGCCAGCAGCCG<br>CGGTAATTCCAGCTCCAATAGCGTATATTAAAGTTGTT<br>GCAGTTAAAAAGCTCGTAGTTGGATTTCTGGCAGGAG<br>CGACCGGTCTCACACTCAGTGCGAGA
Chrysochromulina (18S rRNA)	(Li et al. 2022)	GGGTAACGGAGAATTAGGGTTCGATTCCGGAGAGGG<br>AGCCTGAGAGATGGCTACCACATCCAAGGAAGGCAG<br>CAGGCGCGTAAATTGCCCGAATCCTGACACAGGGAG<br>GTAGTGACAAGAAATAACAATACAGGGCTTCTAAAGT<br>CTTGTAATTGGAATGAGTACAATTTACATCTCTTCACG<br>AGGATCAATTGGAGGGCAAGTCTGGTGCCAGCAGCC<br>GCGGTAATTCCAGCTCCAATAGCGTATATTAAAGTTGT<br>TGCAGTTAGAACGCTCGTAGTCGGATTTCGGGGCGGG<br>TCGACCGGTCTGCCGATGGGTACGCACTGGCCGACG<br>CGTCCTTCCTTCCGGAGACCGTCTCACTCTTAACTGA<br>GCGGAGGCGGGAGACGGAACGTTTACTTTGAAAAAA<br>TCAGAGTGTTTCAAGCAGGCAGCTCGCTCTTGCATGG<br>ATTAGCATGGGATAATGAAATAGGACTTTGGTGCTATT<br>TTGTTGGTTTCGAACACCGAAGTAATGATGAGAAGG<br>GACAGTCAGGGGCACTCGTATTCCGCCGAGAGAGGT<br>GAAATTCTCAGACCAGCGGAAGACGAACCACTGCGA<br>AAGCATTTGCCAGGGATGTTTTCACTGATCAAGAACG<br>AAAGTTAGGGGATCGAAGACGATCAGATACCGTCGTA<br>GTCTTAACCATAAACCATGCCGACTAGGGATTGGAGG<br>ATGTTCACTTATTGACTCCTTCAGCACCTTACGGGAA<br>ACTAAAGTCTTTGGGT
SAR11 (16S rRNA)	(Suzuki et al. 2001)	CGTGAGTGAAGAAGGCCTTTGGGTTGTAAAGCTCTTT<br>CGTCGGGGAAGAAAATGACTGTACCCGAATAAGAAG<br>GTCCGGCTAACTTCGTGCCAGCAGCCGCGGTAATACG<br>AAGGGACCTAGCGTAGTTCGGAATTACTGGGCTTAAA<br>GAGTTCGTAGGTGGTTGAAAAAGTTGGTGGTGAAAT<br>CCCAGAGCTTAAC
Prochlorococcus (eMIT9312 16-23S ITS rRNA)	(Zinser et al. 2006)	GGCGATGAAGGACGTGGTTACCTGCGATAAGTCTCGG<br>GGAGTTGGAAGCACACTTTGATCCGGGAATTTCCGA<br>ATGGGGCAACCCCATGTACGGCCAACTGAATATATAG<br>GTTGGTGCGAGCTAACCCAGCGAAC
Synechococcus (Clade 2 16-23S ITS rRNA)	(Ahlgren and Rocap 2012)	GGAGTTGAGCTCGATTGGAGTGTGATTTAGATGTGTC<br>CGCTGGATGAACCCAGCTTCCTGTCATTCCAAGTTTT<br>AGGTTTAAGCCTTTAATTTGGTTGATAAGATGCTGGG<br>CTCAAATCAAATTAACAAGCAATTGATGATTTGATTTG<br>AAGTTCTAGCAGAACCTTGACAACTGCATAGGTGAA<br>GTCTGGAAAAACAAAGCATCTTACAGATGCATT
Crocosphaera (*nifH*)	(Moisander et al. 2010)	CCCGTTTAATCCTCAACTGTAAAGCTCAGGTAACTGT<br>ATTACACTTAGCTGCTGAAATGGGTTCTGTTGAAGAC<br>TTAGAACTCGAAGACGTAATGCTCGAAGGGTTTGAA<br>GGCATCAAGTGTGTAGAATCTGGTGGTCCTGAGCCTG<br>GAGTTGGTTGTGCTGGTCGTGGTATTATCACCTCCATC<br>AACTTCCTAGAAGAAGAAGGAGCTTACGAAGACTTA<br>GAATTCGTATCCTACGACGTATTAG

**Supplemental Table 2.** Values and references for biomass of each microbial prey type.		
Organism	Carbon quota (pg C per cell)	Reference
Pigmented picoeukaryotes (PPE; flow cytometry detected but taxonomy not discerned)	1.0038	(Ribalet et al. 2019)
SAR11	0.0119	(Cermak et al. 2017)
Prochlorococcus (eMIT9312 ecotype)	0.06	(Cermak et al. 2017)
Synechococcus (WH8102)	0.261	(Ribalet et al. 2019)
Crocosphaera (average large and small)	5.65	(Wilson et al. 2017)

Supplemental Figure 1. *Pegea confoederata* (top 4 panels) and *Salpa maxima* (bottom 4 panels) feeding raw data for incubations at all timepoints, controls, and animals.

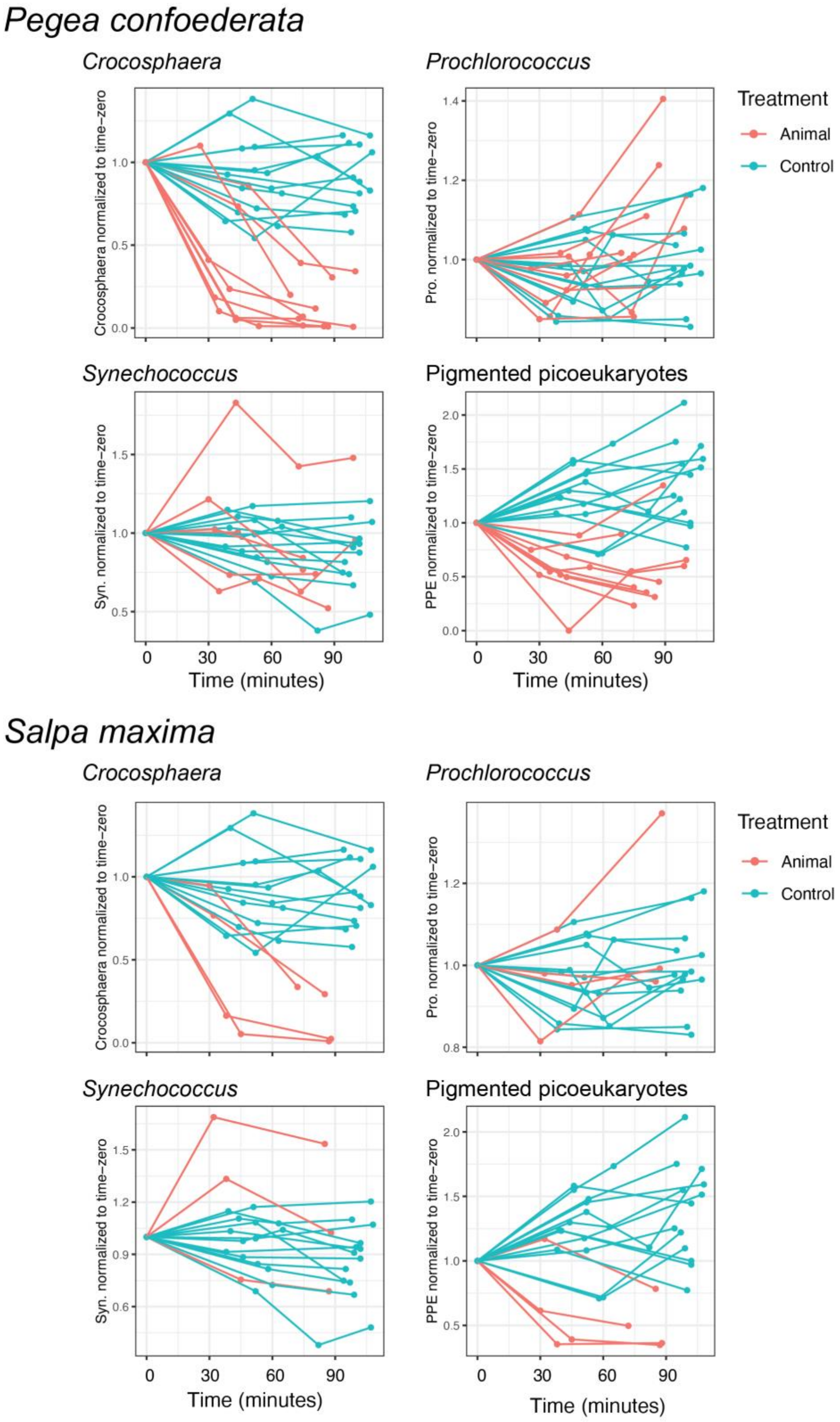

Supplemental Figure 2. Relationship between initial prey concentration (x-axis) and clearance rate (y-axis), with each prey type indicated by color. Abbreviations: PPE (pigmented picoeukaryotes).

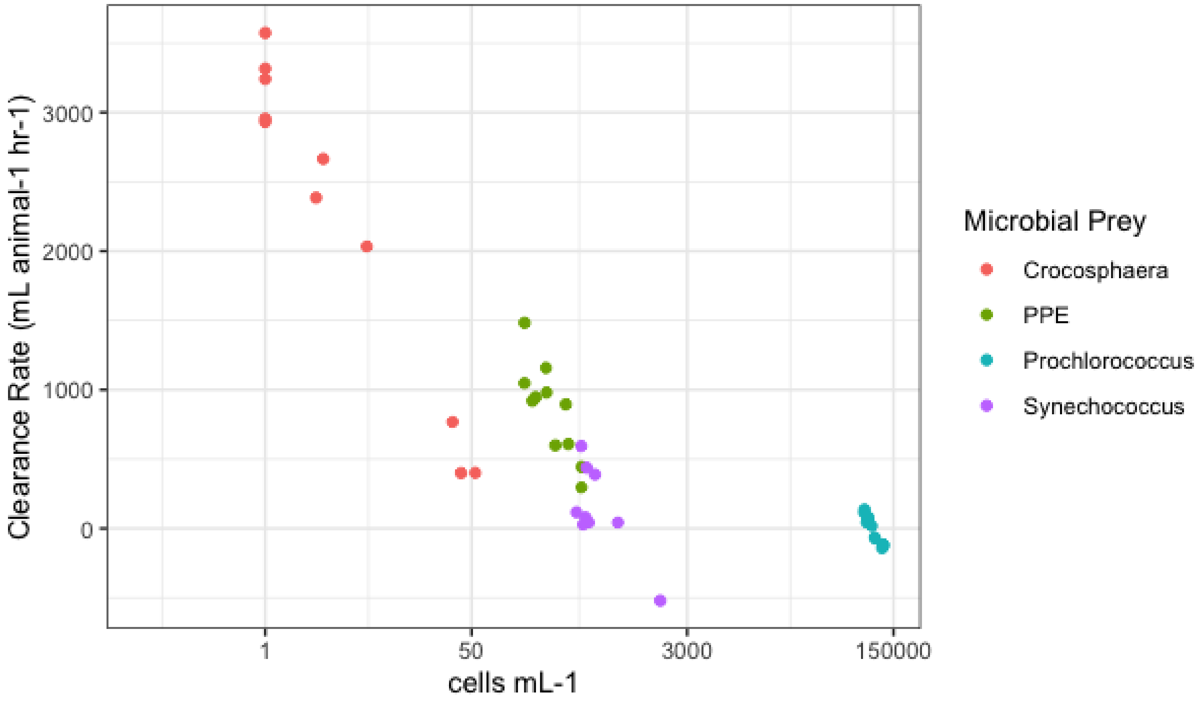

Supplemental Figure 3. Predictions from *P. confoederata* particle encounter models. A) Predicted particle capture efficiency percentage. B) Number of cells per second of the different prey lineages predicted to be captured by the two different particle encounter models (direct interception vs. simple sieving), given the observed cell concentrations in the system (Figure 1).

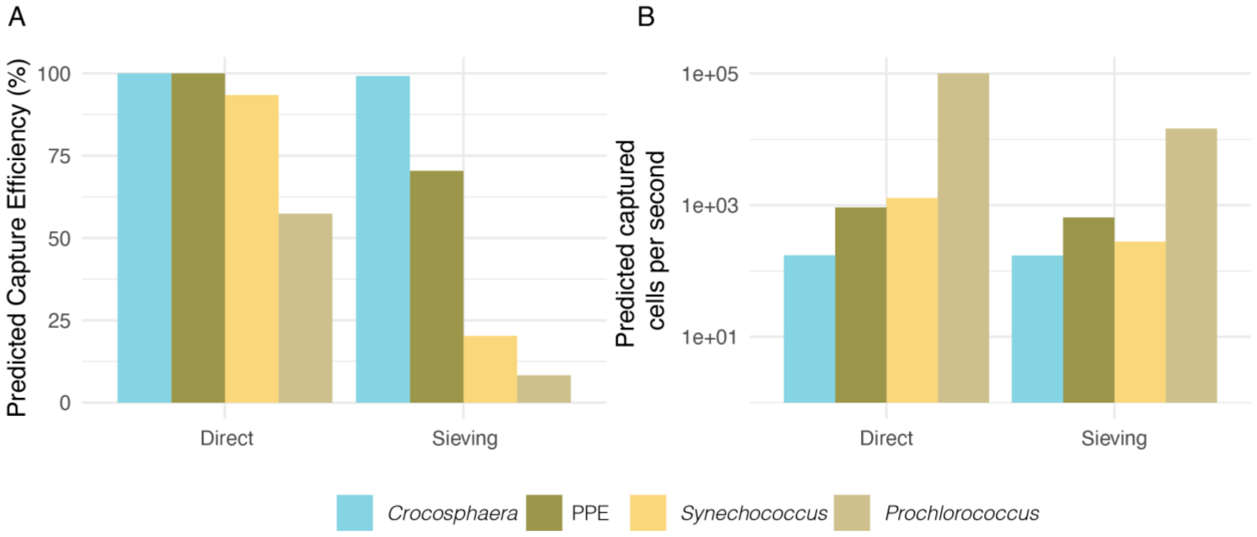

Supplemental Figure 4. Forward light scatter determined by flow cytometry was used to quantify the median cell size of each phytoplankton population using a Mie-based model. A) Histograms of relative forward light scatter for each phytoplankton population and the number of cells (n) measured in a representative sample of the prey field. B) Predicted median diameter of phytoplankton cells based on their relative forward light scatter using a Mie-based model (red and black lines) derived from calibration of the particular BD Biosciences Influx flow cytometer instrument used with polystyrene bead standards, following Ribalet et al. (2019).

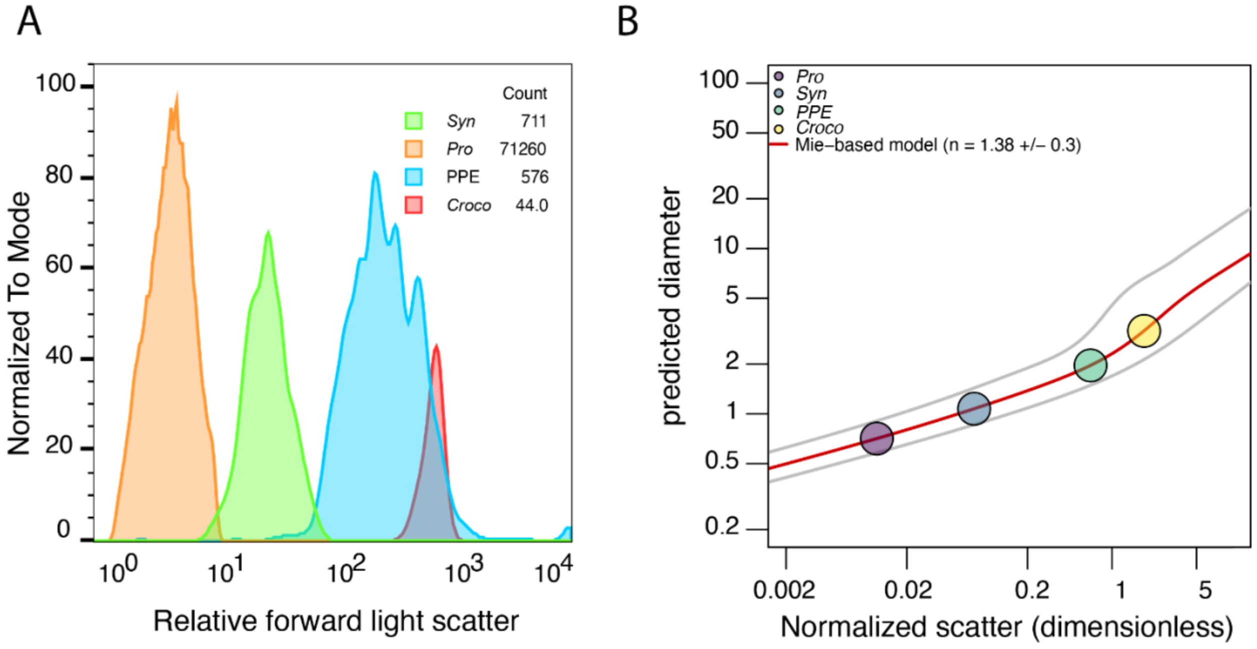

Supplemental Figure 5. Relationship between predicted diameter of phytoplankton based on forward light scatter (x-axis) and clearance rate (y-axis), with each prey type indicated by color. Abbreviations: PPE (pigmented picoeukaryotes).

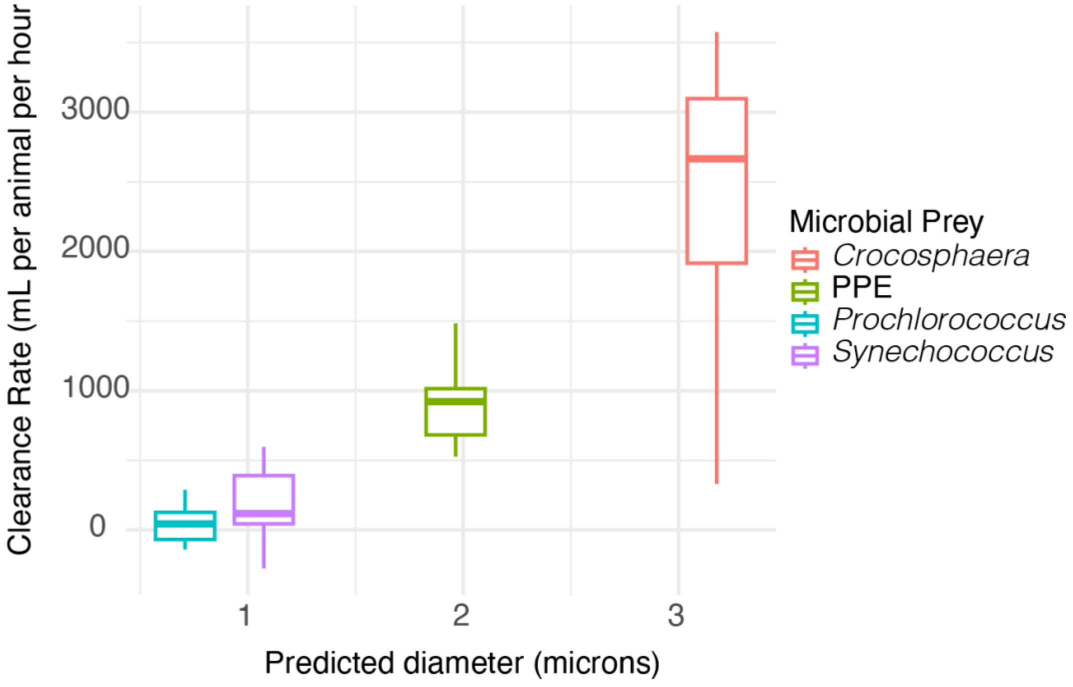

Supplemental References

Ahlgren, Nathan A., and Gabrielle Rocap. 2012. "Diversity and Distribution of Marine Synechococcus: Multiple Gene Phylogenies for Consensus Classification and Development of qPCR Assays for Sensitive Measurement of Clades in the Ocean." *Frontiers in Microbiology* 3 (JUN): 213. https://doi.org/10.3389/fmicb.2012.00213.

Cermak, Nathan, Jamie W. Becker, Scott M. Knudsen, Sallie W. Chisholm, Scott R. Manalis, and Martin F. Polz. 2017. "Direct Single-Cell Biomass Estimates for Marine Bacteria via Archimedes' Principle." *The ISME Journal* 11 (3): 825–28. https://doi.org/10.1038/ismej.2016.161.

Frischer, Marc E., Christy A. Sanchez, Tina L. Walters, Megan E. Thompson, LaGina M. Frazier, and Gustav -A. Paffenhöfer. 2014. "Reliability of qPCR for Quantitative Gut Content Estimation in the Circumglobally Abundant Pelagic Tunicate Dolioletta Gegenbauri (Tunicata, Thaliacea)." *Food Webs* 1 (1): 18–24. https://doi.org/10.1016/j.fooweb.2014.11.001.

Li, Qian, Kyle F. Edwards, Christopher R. Schvarcz, and Grieg F. Steward. 2022. "Broad Phylogenetic and Functional Diversity among Mixotrophic Consumers of Prochlorococcus." *The ISME Journal* 16 (6): 1557–69. https://doi.org/10.1038/s41396-022-01204-z.

Moisander, Pia H, Roxanne A Beinart, Ian Hewson, Angelicque E White, Kenneth S Johnson, Craig A Carlson, Joseph P Montoya, and Jonathan P Zehr. 2010. "Unicellular Cyanobacterial Distributions Broaden the Oceanic N2 Fixation Domain." *Science* 327 (5972): 1512–14. https://doi.org/10.1126/science.1185468.

Ribalet, François, Chris Berthiaume, Annette Hynes, Jarred Swalwell, Michael Carlson, Sophie Clayton, Gwenn Hennon, et al. 2019. "SeaFlow Data v1, High-Resolution Abundance, Size and Biomass of Small Phytoplankton in the North Pacific." *Scientific Data* 6 (1): 277. https://doi.org/10.1038/s41597-019-0292-2.

Suzuki, Marcelino T., Oded Béjà, Lance T. Taylor, and Edward F. DeLong. 2001. "Phylogenetic Analysis of Ribosomal RNA Operons from Uncultivated Coastal Marine Bacterioplankton." *Environmental Microbiology* 3 (5): 323–31. https://doi.org/10.1046/j.1462-2920.2001.00198.x.

Walters, Tina L., Lauren M. Lamboley, Natalia B. López-Figueroa, Áurea E. Rodríguez-Santiago, Deidre M. Gibson, and Marc E. Frischer. 2019. "Diet and Trophic Interactions of a Circumglobally Significant Gelatinous Marine Zooplankter, Dolioletta Gegenbauri (Uljanin, 1884)." *Molecular Ecology* 28 (2): 176–89. https://doi.org/10.1111/mec.14926.

Wilson, Samuel T., Frank O. Aylward, Francois Ribalet, Benedetto Barone, John R. Casey, Paige E. Connell, John M. Eppley, et al. 2017. "Coordinated Regulation of Growth, Activity and Transcription in Natural Populations of the Unicellular Nitrogen-Fixing Cyanobacterium Crocosphaera." *Nature Microbiology* 2 (9): 17118. https://doi.org/10.1038/nmicrobiol.2017.118.

Zinser, Erik R, Allison Coe, Zackary I Johnson, Adam C Martiny, Nicholas J Fuller, David J Scanlan, and Sallie W Chisholm. 2006. "Prochlorococcus Ecotype Abundances in the North Atlantic Ocean As Revealed by an Improved Quantitative PCR Method." *Applied and Environmental Microbiology* 72 (1): 723 LP – 732. https://doi.org/10.1128/AEM.72.1.723-732.2006.
